# The association between smartphone addiction and self-esteem among physical education undergraduate students: The chain-mediating roles of professional identity and meaning in life

**DOI:** 10.1371/journal.pone.0337908

**Published:** 2026-01-02

**Authors:** Pu Sun, Mingyue Cui, Xi Chen, Ling Yan

**Affiliations:** Department of Psychology and Education, Capital University of Physical Education and Sports, Beijing, China; Tianjin University, CHINA

## Abstract

This study investigated the associations between smartphone addiction and self-esteem among undergraduate Physical Education students, focusing on the mediating roles of professional identity and meaning in life. A total of 695 undergraduate students majoring in Physical Education were recruited through a convenience sampling method from a sports university in Beijing. Participants completed the Smartphone Addiction Scale, the Professional Identity Scale for Pre-service Teachers, the Meaning in Life Questionnaire, and the Rosenberg Self-Esteem Scale. Structural equation modelling (SEM) was conducted in Mplus 8.3 to test multiple and chain mediation effects. Model fit indices indicated an acceptable fit (χ²/df = 4.43, CFI = 0.96, TLI = 0.93, RMSEA = 0.08). The results showed that smartphone addiction was significantly and negatively associated with professional identity (*β* = –0.42, 95% CI [–0.51, –0.32], *p* < 0.001) and meaning in life (*β* = –0.29, 95% CI [–0.38, –0.19], *p* < 0.001), both of which were positively associated with self-esteem (professional identity: *β* = 0.36, 95% CI [0.25, 0.46], *p* < 0.001; meaning in life: *β* = 0.31, 95% CI [0.21, 0.42], *p* < 0.001). Smartphone addiction was also directly and negatively associated with self-esteem (*β* = –0.27, 95% CI [–0.37, –0.18], *p* < 0.001). Mediation analyses further indicated that professional identity and meaning in life independently and sequentially mediated this association, forming a significant chain-mediating pathway (total indirect effect = –0.047, 95% CI [–0.071, –0.029]). The final model accounted for 38.2% of the variance in self-esteem (*R*² = 0.382). These findings highlight the psychological mechanisms linking smartphone addiction and self-esteem, offering evidence-based implications for interventions addressing smartphone overuse among Physical Education undergraduates.

## 1 Introduction

Self-esteem is defined as an individual’s subjective evaluation of their own self-worth, and is considered a core component of the self-system [1]. Previous research has shown that self-esteem is closely associated with emotional well-being and psychological adjustment, including anxiety, depression and sensitivity to interpersonal relationships [2]. Furthermore, self-esteem plays a pivotal role in individuals’ career choices and development. Those with high self-esteem are more likely to take a proactive approach to career exploration and decision-making, and tend to experience greater career growth thanks to enhanced self-efficacy and emotional resilience. In contrast, individuals with low self-esteem often struggle with career planning and lack initiative, which can hinder their professional development [3].

For students majoring in Physical Education, becoming a physical education (PE) teacher is one of the most common career trajectories. However, PE teachers face multiple professional challenges, such as the marginalisation of physical education in schools, the uneven allocation of curriculum resources and persistent social stereotypes regarding the professionalism of PE teaching [4]. For undergraduates in the early stages of forming their professional identity, having a higher level of self-esteem can make them feel more professionally fulfilled and responsible, strengthen their commitment to a teaching career and help them to build supportive networks of colleagues — thus equipping them better to deal with the demands of professional socialisation.

Compared with students in other disciplines, PE majors may be more dependent on smartphones. Their coursework and training are often constrained by practical conditions, making smartphones an important tool for accessing information, maintaining social connections and engaging in leisure activities. However, PE programmes emphasise physical activity and practical training, so excessive smartphone use could disrupt the balance between academic engagement and a healthy lifestyle. This poses risks to professional development and mental health. Previous studies have shown that smartphone addiction is linked to sleep disturbances, attention problems, and reduced self-control [5]. These issues are particularly pertinent among PE students, for whom physical competence and psychological stability are fundamental requirements. Consequently, this group may be particularly susceptible to the adverse effects of smartphone addiction on mental health and self-esteem.

Although previous studies have examined the associations between self-esteem, mental health, and academic achievement among university students, the mechanisms underlying the development of self-esteem among students majoring in Physical Education remain underexplored. In particular, with the growing penetration of digital media into students’ daily lives, smartphone addiction has emerged as a significant behavioral risk factor that may undermine psychological well-being and personal development [6]. However, limited research has investigated how smartphone addiction may impair self-esteem through internal psychological pathways, and the theoretical underpinnings of such associations remain insufficiently articulated. In particular, for students majoring in Physical Education, although they are expected to undertake the critical mission of promoting youth health and physical education in the future, their professional identity is still in the process of formation, and their psychological resources are more susceptible to external influences. Therefore, it is especially important to further investigate how smartphone use affects the core self-system within this population.

Furthermore, two psychological constructs — professional identity and meaning in life — have recently been the focus of much youth development research, as they are considered to be important links between behaviour and self-evaluation. However, the role of these constructs as mediators in the relationship between smartphone addiction and self-esteem has rarely been examined, particularly in relation to pre-service physical education teachers.

In order to address these gaps, the present study draws on Self-Determination Theory (SDT) and Social Cognitive Theory (SCT) in order to construct a structural model in which smartphone addiction influences self-esteem by way of the sequential mediators of professional identity and meaning in life. The study aims to provide a more nuanced understanding of the impact of digital behaviour on core aspects of the self-system, offering theoretical and practical insights into enhancing the professional identity and psychological well-being of Physical Education students.

## 2 Literature review

### 2.1 Theoretical framework

#### 2.1.1 Self-determination theory.

Self-Determination Theory, developed by Deci and Ryan, is a motivational process theory concerning human behaviour that is driven by a sense of autonomy. It has been widely applied in the fields of education, mental health and behavioural motivation to explain individuals’ actions. SDT posits that psychological well-being and positive behaviours depend on the satisfaction of three basic psychological needs: autonomy, competence and relatedness. Autonomy refers to a sense of volition and choice in one’s actions, while competence reflects perceptions of capability and experiencing mastery when accomplishing tasks. Relatedness, meanwhile, denotes feelings of belonging and emotional connection in social interactions.Satisfying these three basic needs leads to stronger intrinsic motivation. In educational settings, this manifests as sustained attention and deep engagement, encouraging active exploration and reflection. This enhances academic achievement and confidence. In the context of work and career development, it is reflected in taking on more responsibility, showing initiative and being creative, which supports the establishment of stable career goals and a professional identity. In daily life, satisfaction of these needs strengthens emotional regulation and interpersonal functioning, enabling greater well-being and social support. Thus, autonomy, competence and relatedness are not only essential for mental health, but also vital psychological resources for personal growth and social adaptation. Conversely, when these needs are persistently thwarted, individuals may gradually lose intrinsic motivation and experience motivational depletion. A lack of autonomy can lead to passivity and feelings of helplessness in academic and life domains, while compromised competence can result in frustration when pursuing and achieving goals, thereby diminishing self-efficacy and self-worth. Deprivation of relatedness can foster feelings of isolation and social alienation [7].

This study conceptualises smartphone addiction as a form of behavioural dysregulation, characterised by an excessive dependence on instant gratification, a diminished sense of control in real life and reduced quality of interpersonal interactions. These features may interfere with the fulfilment of basic psychological needs in several ways. Compulsive smartphone use can limit an individual’s freedom of choice and behavioural autonomy, putting them in a passive and reactive state that undermines their sense of self-directed agency. It may also reduce engagement in real-world goal pursuit and accomplishment, thus impairing the development of competence. Furthermore, reliance on virtual communication may replace meaningful face-to-face relationships, thereby reducing the sense of belonging.

Chronic frustration of the three psychological needs of autonomy, competence and relatedness may damage individuals’ intrinsic motivational system, weakening their drive for career development and hindering the formation of professional identity. At the same time, their capacity for emotional connection and their ability to perceive value in meaning-making may be suppressed, which undermines their sense of purpose in life. Ultimately, prolonged deprivation of these psychological resources can lead to lower self-evaluation and diminished self-esteem.

#### 2.1.2 Social cognitive theory.

Social Cognitive Theory, proposed by psychologist Albert Bandura, posits a triadic reciprocal relationship among personal factors, behavior, and the social environment. In this framework, individuals’ thoughts and beliefs are shaped by their external environment, yet they also actively influence and reshape it through their own actions. Unlike traditional perspectives that view individuals as passive responders to external forces, SCT emphasises that individuals are active beings who are goal-directed, capable of regulating their behaviour and engaged in reflective meaning-making throughout development [8]. Through ongoing interactions with their social environment, individuals receive continuous feedback from others and society. This gradually helps them to construct their understanding of occupational roles, as well as their sense of life purpose. This process involves external social inputs and internal mechanisms such as self-observation, self-judgement and self-reflection. Through these mechanisms, individuals transform social experiences into an understanding of their self-worth and the meaning of life, which in turn shapes their self-esteem [9].

From the perspective of SCT, individuals develop a professional identity and a sense of purpose in life by absorbing feedback from their surroundings and developing value-based self-perceptions. These experiences provide cognitive cues that trigger internal evaluative mechanisms, shaping and regulating self-esteem in the process. Professional identity reflects the value-based construction of one’s occupational self, while meaning in life represents a coherent understanding of one’s existence and purpose. Individuals with a strong professional identity and clear life goals are more likely to have a stable and integrated sense of self, fostering positive self-evaluation and higher self-esteem.

However, smartphone addiction can interfere with this process by immersing individuals in virtual environments and encouraging them to avoid real-life challenges. This reduces their investment in occupational goals and undermines the development of a professional identity. It may also limit opportunities for self-reflection and meaning-making, blur life goals and reduce the capacity for self-regulation. Ultimately, these factors can erode positive self-perception and lead to diminished self-esteem.

### 2.2 Smartphone addiction and self-esteem

With the rapid increase in smartphone usage, smartphone addiction has become a widely discussed behavioral concern. It is typically characterized by excessive reliance on smartphones and the inability to control their usage [6]. Empirical studies have shown that smartphone addiction is closely associated with various negative psychological outcomes, including depression, anxiety, loneliness, sleep disturbances, and reduced well-being [10].

Among university students, researchers have found a significant negative correlation between smartphone addiction and self-esteem [11]. Excessive smartphone use may impair self-regulation, hinder the completion of real-life tasks and lead to feelings of helplessness and frustration, thereby reducing perceived self-efficacy and self-worth. On the other hand, frequent engagement with social media platforms may encourage social comparison, exposing individuals to others’ selectively presented successes and attractiveness. This can evoke feelings of relative deprivation and self-doubt, further undermining self-esteem.

While many studies support a negative relationship between smartphone addiction and self-esteem, some findings suggest that the association is not always significant [12]. Such inconsistencies may be due to differences in research methods, sample characteristics or cultural contexts. Further investigation is therefore needed to clarify the nature of this relationship across different populations and settings.

### 2.3 The mediating role of professional identity in the relationship between smartphone addiction and self-esteem

Professional identity in teaching refers to individuals’ cognitive, emotional and behavioural identification with the teaching profession. Shaped by one’s educational environment, prior experiences, and participation in learning communities, it is considered a key psychological construct in the professional socialisation process of pre-service teachers [13]. As an internal driver of career development, professional identity not only influences an individual’s sense of professional belonging and responsibility but also serves as a strong predictor of career adaptability and mental well-being.

Recent studies have found that smartphone addiction negatively predicts identity development [14]. This may be due to the adverse effects of excessive smartphone use, such as impaired attention, reduced self-control and a diminished capacity for information processing. These factors collectively weaken an individual’s engagement with real-world goals, including academic learning and the internalisation of occupational roles. For students of Physical Education, prolonged smartphone use may hinder the acquisition of subject-specific knowledge and practical teaching skills. More critically, it may prevent the development of a meaningful teaching identity.

Meanwhile, a growing body of evidence supports a significant positive association between professional identity and self-esteem [15]. When individuals have a clear and positive understanding of their occupational roles, they are more likely to demonstrate sustained professional engagement and motivation to achieve. This fosters emotional stability and self-worth, and contributes to enhanced self-esteem through positive feedback and a sense of fulfilment gained through practical experience [16].

### 2.4 The mediating role of meaning in life in the relationship between smartphone addiction and self-esteem

Meaning in life refers to individuals’ perception of life’s value and purpose [17], and serves as a key indicator of psychological well-being and positive adaptation. Studies have shown that people who find life meaningful are more likely to experience a sense of direction and inner purpose, accompanied by more frequent positive emotions and stable self-affirmation [18]. Conversely, a lack of meaning in life has been linked to psychological distress, including depression, loneliness, and self-devaluation [17].

Recent empirical evidence suggests that maladaptive smartphone use is negatively associated with individuals’ sense of meaning in life [19]. This may be due to behavioral patterns involving compulsive scrolling, social comparison, and pursuit of instant gratification, which can draw individuals away from meaningful real-world engagement, goal-setting, and value-based reflection. According to SDT, smartphone addiction may compromise individuals’ autonomy, reduce their sense of competence in everyday achievements, and hinder real interpersonal relationships, all of which can significantly diminish their perceived meaning in life [20]. Together, these mechanisms may prevent individuals from setting clear life goals or affirming their existential value. Furthermore, previous studies have consistently identified a strong positive correlation between life meaning and self-esteem [21]. People who have a clear understanding of their life goals and feel that their life has meaning are more likely to have a positive self-perception and higher self-esteem.

### 2.5 The chain-mediating role of professional identity and meaning in life

For students majoring in Physical Education, professional identity is a central construct in the process of professional socialisation and a critical component of self-system stability. Previous research has shown that pre-service teachers with a stronger professional identity tend to report higher levels of academic and life satisfaction, greater clarity in their career goals, and a stronger sense of self-worth. They are also more likely to view the teaching profession as an integral part of their identity, developing a clearer sense of purpose and social responsibility, thus enhancing their overall experience of meaning in life [13,22]. In developmental terms, the establishment of professional identity often precedes the deepening of existential meaning. That is, a strong sense of value and direction within one’s occupational role may serve as a foundation for forming a broader sense of meaning in life. For students majoring in Physical Education, developing a professional identity can foster confidence and commitment to a teaching career, and may indirectly enhance their sense of purpose and self-worth by reinforcing a sense of mission and social contribution.

However, smartphone addiction may have a negative impact on this process. Studies indicate that excessive use of digital media can reduce participation in real-life professional activities and hinder engagement with exploring the role of a teacher, thereby weakening professional identity [14]. A weakened sense of professional identity can undermine an individual’s ability to reflect on their life goals and affirm their existential value. This can ultimately lead to a disruption in meaning-making and a deterioration in self-esteem.

Therefore, it is reasonable to assume that smartphone addiction may undermine professional identity, thereby reducing meaning in life and eventually impairing self-esteem. This suggests that professional identity and meaning in life may act as sequential mediators, forming a chain from social role cognition to existential valuation.

Given the distinctive educational and professional context of Physical Education majors, it is essential to examine how these mechanisms operate within this population. Therefore, the following hypotheses are proposed:

***H1:*** Smartphone addiction is significantly negatively associated with self-esteem among students majoring in Physical Education.

***H2:*** Professional identity mediates the relationship between smartphone addiction and self-esteem among students majoring in Physical Education.

***H3:*** Meaning in life mediates the relationship between smartphone addiction and self-esteem among students majoring in Physical Education.

***H4:*** Professional identity and meaning in life jointly mediate the relationship between smartphone addiction and self-esteem among students majoring in Physical Education through a chain-mediating effect.

## 3 Materials and methods

### 3.1 Participants

A convenience sampling method was used to recruit undergraduate students majoring in Physical Education from a sports university in Beijing, based on the accessibility of this population and feasibility considerations. The inclusion criteria were: currently enrolled publicly funded Physical Education undergraduates (pre-service teacher education students) at the university. Students who were on academic leave or enrolled in postgraduate programs (master’s or doctoral) were excluded.

In total, 792 students were invited to participate, and 695 valid questionnaires were collected via the online survey platform Wenjuanxing, yielding an effective response rate of 87.75%. Among the participants, 521 were male (74.96%) and 174 were female (25.04%), with an average age of 19.98 years (SD = ±1.19). Detailed demographic information of the participants is presented in Table 1. Recruitment and data collection were conducted concurrently over an 11-day period (March 11–21, 2025). Faculty advisors assisted the research team in disseminating the survey link and QR code to eligible students through official class groups. Participants completed the survey on a rolling basis as they received the link; thus, recruitment and data collection occurred simultaneously during the study window. No monetary or academic incentives were provided.

**Table 1:** Demographic characteristics of participants (*N* = 695).

Variable	Category	*n*	%	M	SD
Gender	Male	521	74.96		
	Female	174	25.04		
Age (years)				19.98	1.19
Grade level	Freshman	125	17.99		
	Sophomore	216	31.08		
	Junior	181	26.04		
	Senior	173	24.89		
Total		695	100		

Values are presented as number of participants (n) and percentage (%) for categorical variables and mean (M) ± standard deviation (SD) for continuous variables.

The online platform was configured such that participants could review all items before submission; however, once they began the survey, the system required completion of all items before the questionnaire could be successfully submitted. This design minimized the occurrence of missing data, and no incomplete responses were recorded in the dataset. The unit of analysis was the individual participant, and the dataset contained no clustering or stratification structure.

The study was approved by the Ethics Committee of Capital University of Physical Education and Sports (Approval No. 2025A052), and institutional permission was obtained from the participating university. In accordance with the approved protocol, all participants were orally informed about the study’s purpose and procedures, and their voluntary completion of the anonymous questionnaire was regarded as provision of verbal informed consent, consistent with prior IRB-approved minimal-risk online survey studies.

### 3.2 Instruments

#### 3.2.1 Short version of the smartphone addiction scale (SAS-SV).

The Short Version of the Smartphone Addiction Scale, developed by Kwon et al. [23], and later translated and validated in Chinese by Zhang et al. [24], was used to assess participants’ tendency toward problematic smartphone use. The scale consists of 10 items rated on a 6-point Likert scale (1 = “Strongly Disagree” to 6 = “Strongly Agree”). Higher total scores indicate greater levels of smartphone addiction. The total score was calculated by summing all item scores. In the present study, Cronbach’s α was 0.944, indicating excellent internal consistency.

#### 3.2.2 Professional identity scale for normal university students (PISNS).

The Professional Identity Scale for Normal University Students, developed by Wang et al. [25] based on the Chinese national teacher education context, was used to assess participants’ sense of professional identity as pre-service teachers. The scale comprises 12 items across four dimensions: professional aspiration and expectation, professional will, professional values, and professional efficacy. Items are rated on a 5-point Likert scale (1 = “Strongly Disagree” to 5 = “Strongly Agree”), with higher scores reflecting stronger professional identity. The total score was computed by summing all item responses. Cronbach’s α for the total scale was 0.907, and for the four subscales were 0.935, 0.836, 0.867, and 0.943, respectively, demonstrating good internal consistency.

#### 3.2.3 Meaning in life questionnaire (MLQ).

The Meaning in Life Questionnaire, developed by Steger et al. [17] and revised for use in Chinese populations by Liu and Gan [26], was used to evaluate participants’ perceived meaning in life. The MLQ contains 10 items covering two subscales: Presence of Meaning (POM) and Search for Meaning (SFM). Responses are rated on a 7-point Likert scale (1 = “Absolutely Untrue” to 7 = “Absolutely True”), with higher scores indicating a stronger sense of life meaning. The total score was computed as the sum of all items. Cronbach’s α for the total scale was 0.915, and for the POM and SFM subscales were 0.945 and 0.831, respectively, reflecting excellent reliability.

#### 3.2.3 Rosenberg self-esteem scale (RSES).

The Rosenberg Self-Esteem Scale, originally developed by Rosenberg [27], and later translated and validated in Chinese by Yang and Wang [28], was employed to assess participants’ global self-esteem. The RSES consists of 10 items, including five positively and five negatively worded items, rated on a 4-point Likert scale (1 = “Completely Untrue” to 4 = “Completely True”). Negatively worded items were reverse-scored before computing the total score, with higher totals indicating higher self-esteem. In this study, Cronbach’s α was 0.792, suggesting acceptable internal consistency.

#### 3.2.4 Data screening and outlier detection.

Before conducting analyses, data were screened to ensure completeness and acceptable distributional properties. Because the online platform required participants to complete all items before submission, there were no missing data. The absolute values of skewness and kurtosis for all observed indicators were below 1.50, suggesting approximate univariate normality. In line with previous robustness checks, three participants whose self-esteem scores exceeded ±3 standard deviations from the mean were examined as potential outliers; their exclusion did not materially affect the results, so they were retained for the final analyses.

### 3.3 Quality control

In this study, the questionnaire was created using the Wenjuanxing online platform, which generated a QR code and an online access link for distribution. Prior to formal data collection, members of the research team conducted a pilot test to verify the clarity, reliability, and completion logic of the questionnaire. Based on the pilot feedback, minor wording adjustments were made to optimize the survey content. During the distribution phase, the research team communicated with faculty advisors, explained the purpose and procedures of the survey in detail, and entrusted them with disseminating the questionnaire link to Physical Education undergraduates in the relevant grade levels.

After data collection, the returned questionnaires were checked for completeness and logical consistency (e.g., response time and pattern) to ensure data quality. Because the online platform required participants to complete all items before submission, no missing or incomplete responses were present, and all valid questionnaires were retained for subsequent analysis. A double-entry verification procedure was performed by two independent researchers during data export to ensure accuracy and reliability.

### 3.4 Data processing

Data analysis was performed using IBM SPSS 29.0 to conduct common method bias testing and partial correlation analysis. Mplus 8.3 was used to construct structural equation models and test the chain mediation effect, with 5,000 bootstrap resamples and a significance level of α = 0.05. The analyses were conducted using the robust maximum likelihood estimator (MLR), and all observed variables were treated as continuous, as the items used 5–7 response categories and showed approximately symmetric distributions. Prior simulation research has demonstrated that treating Likert-type items with five or more response categories as continuous under robust ML estimation yields unbiased parameter estimates and valid inferences [29].

## 4 Results

### 4.1 Common method bias control and testing

All study variables were measured through self-report questionnaires, which may introduce potential common method bias (CMB). To address this issue, both procedural and statistical controls were implemented. During data collection, participants were assured of anonymity and confidentiality, and were informed that their responses would be used solely for scientific research purposes. In addition, reverse-coded items were incorporated across several scales to minimize response bias and acquiescence effects.

To statistically examine the presence of CMB, Harman’s single-factor test was first conducted. The unrotated exploratory factor analysis extracted eight factors with eigenvalues greater than 1, and the first factor accounted for 31.75% of the total variance—below the commonly accepted threshold of 40%. Subsequently, a single-factor confirmatory factor analysis (CFA) was performed by loading all items onto one latent factor. The model fit was poor (χ²/df = 48.99, CFI = 0.74, GFI = 0.80, AGFI = 0.67, NFI = 0.73, RMSEA = 0.19), indicating that a single latent factor could not adequately explain the data structure.

To further validate these findings, a latent method factor was incorporated into the baseline measurement model, with each item allowed to load on both its theoretical construct and the method factor. The method factor was constrained to be uncorrelated with all substantive latent variables, and its variance was fixed to 1. The inclusion of this factor resulted in a substantially poorer fit (χ²/df = 42.98, CFI = 0.34, TLI = 0.22, RMSEA = 0.30), compared with the baseline measurement model (χ²/df = 4.43, CFI = 0.96, TLI = 0.93, RMSEA = 0.08).

Taken together, the results from these procedural controls and multiple statistical tests consistently indicate that common method bias was not a serious concern in this study.

### 4.2 Descriptive statistics and partial correlation analysis

After controlling for gender, age, and grade level [30–32], the results of the partial correlation analysis among the variables are presented in Table 2. Smartphone addiction was significantly negatively correlated with professional identity (*r* = −0.11), meaning in life (*r* = −0.16), and self-esteem (*r* = −0.40). Professional identity was significantly positively correlated with meaning in life (*r* = 0.64) and self-esteem (*r* = 0.49). In addition, meaning in life was also significantly positively correlated with self-esteem (*r* = 0.46).

**Table 2:** Descriptive statistics and partial correlations among variables (*N* = 695).

Variable	M	SD	1	2	3	4
1. Smartphone addiction	34.62	11.61	—			
2. Professional identity	49.55	6.99	−0.11**	—		
3. Meaning in life	54.72	9.87	−0.16***	0.64***	—	
4. Self-esteem	29.82	4.06	−0.40***	0.49***	0.46***	—

Partial correlations were computed controlling for gender, age, and grade level.

**p < 0.01. ***p < 0.001.

These results indicate close relationships among smartphone addiction, professional identity, meaning in life, and self-esteem, providing empirical support for subsequent hypothesis testing. Furthermore, to ensure the absence of multicollinearity among the study variables, variance inflation factors (VIFs) and tolerance statistics were examined in SPSS 29.0. The VIF values ranged from 1.03 to 1.64, and tolerance values ranged from 0.61 to 0.97, all within acceptable thresholds (VIF < 5; tolerance > 0.10), indicating no multicollinearity concerns.

### 4.3 Measurement model

Before testing the hypothesized mediation model, a CFA was performed using Mplus 8.3 to evaluate the adequacy of the joint measurement model, which included four latent constructs: smartphone addiction (SAS-SV), professional identity (PISNS), meaning in life (MLQ), and self-esteem (RSES). All standardized factor loadings were significant (*p* < 0.001) and exceeded 0.60, indicating satisfactory convergent validity. The measurement model demonstrated an acceptable fit to the data, χ²/df = 4.43, GFI = 0.95, AGFI = 0.90, CFI = 0.96, TLI = 0.93, IFI = 0.96, RMSEA = 0.08 (90% CI [0.071, 0.101]). These findings support the distinctiveness of the latent constructs and confirm that the overall measurement structure was adequate for subsequent SEM analysis.

### 4.4 Mediation effect analysis

A mediation analysis was conducted with smartphone addiction as the independent variable, self-esteem as the dependent variable, and professional identity and meaning in life as mediating variables. The results showed that the total effect of smartphone addiction on self-esteem was significant (*B* = −0.140, *p* < 0.001; *β* = −0.332). After controlling for the mediators, the direct effect of smartphone addiction on self-esteem remained significant (*B* = −0.116, *p* < 0.001; *β* = −0.286), indicating that the negative effect of smartphone addiction on self-esteem was partially mediated, while a direct effect still existed independently.

The model fit indices were satisfactory, with the following values: χ²/df = 4.43, GFI = 0.95, NFI = 0.94, RFI = 0.91, IFI = 0.96, TLI = 0.93, CFI = 0.96, RMSEA = 0.08, and standardized RMR = 0.05. According to commonly accepted criteria (CFI/TLI ≥ 0.90 and RMSEA ≤ 0.08 [33,34]), these results indicate that the proposed model demonstrates an acceptable fit and adequately represents the observed data.

**Fig 1:**
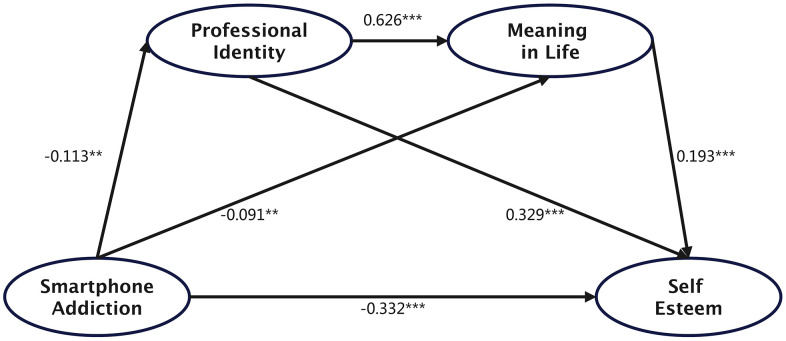
Path diagram of the chain mediation model.

The bias-corrected non-parametric percentile Bootstrap method was used to test the significance of the mediation effect, with 5,000 resamples drawn from the original dataset. Further analysis revealed three significant indirect paths through which smartphone addiction influenced self-esteem (see Fig 1 and Table 3):

**Table 3:** Direct, indirect, and total effects in the multiple mediator model.

Path Type	Effect Value	Total Effect	Indirect Effect	Interpretation of Statistical Significance
Direct effect	−0.116	82.86%	–	Primary effect path
Total indirect effect	−0.024	17.14%	100%	Overall importance of mediation mechanisms
Professional identity only	−0.013	9.29%	54.17%	Most prominent mediating path
Meaning in life only	−0.006	4.29%	25.00%	Secondary mediating path
Chain mediation	−0.005	3.57%	20.83%	Complementary mediation mechanism

Smartphone addiction → Professional identity → Self-esteem:

Smartphone addiction significantly decreased professional identity (*B* = −0.057, *p* = 0.012; *β* = −0.113), and professional identity positively predicted self-esteem (*B* = 0.229, *p* < 0.001; *β* = 0.329). The indirect effect was −0.013 (95% CI [−0.024, −0.002]; *β *= −0.037).

Smartphone addiction → Meaning in life → Self-esteem:

Smartphone addiction significantly reduced meaning in life (*B* = −0.078, *p* = 0.001; *β* = −0.091), which in turn positively predicted self-esteem (*B* = 0.079, *p* < 0.001; *β* = 0.193). The indirect effect was −0.006 (95% CI [−0.012, −0.002]; *β* = −0.018).

Smartphone addiction → Professional identity → Meaning in life → Self-esteem (Chain mediation):

Professional identity significantly enhanced meaning in life (*B* = 1.061, *p* < 0.001; *β* = 0.626). The indirect chain effect was −0.005 (95% CI [−0.010, −0.001]; *β* = −0.014).

The final model explained 38.2% of the variance in self-esteem (*R*² = 0.382), and the Bootstrap results supported the robustness of all mediation paths (as all 95% CIs excluded zero). In addition, pairwise comparisons of the indirect effects showed that the mediation effect through professional identity alone was significantly greater than that of the chain mediation path (*p* = 0.047), whereas the differences between the other two paths were not statistically significant (*p* > 0.05).

These results suggest that smartphone addiction undermines individuals’ self-esteem indirectly by reducing their sense of professional identity and meaning in life, with the mediating role of professional identity being particularly prominent.

Additionally, to further confirm the robustness of the findings, a sensitivity analysis was performed using an alternative operationalization approach. The mediation model was re-estimated with mean-scale composite scores in place of latent variables, and the pattern and significance of all direct and indirect effects remained consistent. This indicates that the results are stable across different measurement specifications.

## 5 Discussion

Self-esteem is a fundamental psychological resource for students majoring in Physical Education, influencing their professional confidence, teaching engagement, social adaptability, and mental well-being. While previous research has primarily focused on the general student population, limited attention has been paid to how discipline-specific factors shape self-esteem development in pre-service PE teachers. To address this gap, the present study examined the associations between smartphone addiction and self-esteem among Physical Education undergraduates, emphasizing the mediating roles of professional identity and meaning in life. Given the cross-sectional design, the findings should be interpreted as associations rather than causal effects. The observed relationships provide contextualized insights into how psychological and behavioral factors may co-vary within this population and offer preliminary directions for enhancing professional identity and psychological well-being in PE teacher training programs.

The results first confirmed Hypothesis 1, indicating that smartphone addiction was significantly and negatively associated with self-esteem among students majoring in Physical Education. This finding is consistent with previous research [35] and adds evidence of the adverse correlates of excessive smartphone use. From a mechanistic perspective, overreliance on smartphones can impair an individual’s ability to self-regulate, amplify feelings of helplessness and self-doubt, and trigger negative emotional responses. These factors can collectively erode the self-evaluation system. Furthermore, the social comparison processes commonly associated with social media, especially upward comparison, can lead to feelings of inadequacy and a sense of detachment from reality, exacerbating declines in self-esteem. However, these patterns should be interpreted as associations rather than causal effects, given the cross-sectional nature of the data. Other unmeasured factors, such as self-control, body image, or perceived social support, might simultaneously influence both smartphone use and self-esteem. The present evidence therefore reflects modest, correlational links, suggesting directions for longitudinal or experimental research to clarify underlying mechanisms.

Mediation analysis further confirmed Hypothesis 2, indicating that professional identity serves as a significant mediator between smartphone addiction and self-esteem. This finding is consistent with previous research [14] and extends current empirical evidence regarding the relationship between media dependency and identity construction. Among Physical Education students, professional identity serves as a psychological foundation for occupational goal orientation and social role recognition, acting as a vital link between external behavioural dysregulation and the internal self-system. This study shows that smartphone addiction can reduce students’ engagement with and understanding of their professional roles, which can lead to a weaker sense of professional identity. Additionally, the results showed that professional identity significantly and positively predicted self-esteem among Physical Education students, in line with prior findings [15]. From a psychological perspective, individuals with a stronger sense of professional identity are more likely to evaluate themselves positively. Through active engagement in teaching practice and social interaction, they receive positive feedback that reinforces their self-worth and enhances their self-esteem. This result highlights the importance of professional identity as a core psychological resource for the career development and personal growth of pre-service PE teachers.

The study also found that meaning in life mediates the relationship between smartphone addiction and self-esteem, supporting Hypothesis 3, indicating that meaning in life acted as an additional indirect pathway between smartphone addiction and self-esteem. Students reporting higher smartphone dependency tended to perceive lower meaning in life, which in turn related to diminished self-esteem. This finding is consistent with previous research [19], which has shown that maladaptive media use is significantly negatively correlated with individuals’ sense of meaning in life. According to the Basic Psychological Needs Satisfaction Model, autonomy, competence, and relatedness contribute to meaning and self-worth; smartphone overuse—often reflecting avoidant or compensatory behavior—may coincide with reduced real-world engagement, thereby limiting fulfillment of these needs [36]. However, smartphone addiction—characterized as an avoidant behavioral pattern—may lead individuals to disengage from real-world responsibilities and deep involvement, thereby weakening their sense of control, achievement, and social connectedness. This, in turn, obstructs the fulfillment of basic psychological needs and reduces overall well-being and perceived life meaning [37]. Moreover, previous studies have shown that meaning in life is a foundational psychological source of self-esteem [21,38]. When individuals are able to assign positive meaning to their life and professional experiences, they are more likely to affirm their self-worth and develop a stable and positive sense of self. Conversely, a lack of meaning in life may lead to blurred goals, self-denial, and emotional emptiness, all of which hinder the development and maintenance of self-esteem [17]. This study empirically verified these relationships within the context of students majoring in Physical Education, further advancing our understanding of the role of meaning in life in the psychological construction of self-esteem.

The study also showed that professional identity and meaning in life jointly and sequentially functioned as indirect pathways linking smartphone addiction to self-esteem, providing partial support for Hypothesis 4. These results suggest a correlational pattern in which higher levels of smartphone addiction were associated with lower professional identity, which in turn related to lower meaning in life and subsequently lower self-esteem. This finding extends existing research on how technology-related behaviors may coincide with variations in both vocational and existential dimensions of the self. Previous research has shown that individuals with higher levels of professional identity tend to demonstrate clearer goal orientation, stronger self-efficacy and greater career commitment during career development. These characteristics are associated with a more stable perception of meaning in social roles and life direction [13]. Conversely, heavier smartphone use may coincide with reduced engagement in professional exploration and preparation, thereby limiting the internalization of professional values and commitment. The present findings therefore illustrate a potential sequential association among professional identity, meaning in life, and self-esteem. However, given the cross-sectional and self-report nature of the data, these results cannot establish temporal ordering or causal direction. Other psychological traits, such as self-regulation, emotional stability, or perceived social support, might also underlie these associations. The chain-mediation evidence should thus be interpreted as exploratory and correlational, providing preliminary insight into how professional identity and meaning in life may co-vary with smartphone use and self-esteem among PE students. From a practical standpoint, although the effect sizes were modest, interventions emphasizing career identity formation and meaning-oriented counseling may be promising directions for future applied work, but further longitudinal or experimental research is required to test effectiveness.

Although this study proposed and tested a mediation model based on SDT and SCT, the findings should be interpreted within the limitations of an observational, cross-sectional design. Alternative explanations are also plausible. For example, it is possible that lower self-esteem leads to greater smartphone use, rather than the other way around. In addition, third variables such as self-control, personality traits, or perceived social support may influence both smartphone addiction and self-esteem simultaneously, thereby producing associations that mimic the proposed mediation pathway. Contextual factors—including academic pressure and the relatively marginalized disciplinary status of Physical Education—may likewise shape students’ self-perceptions independently of smartphone use. To clarify these possibilities, future research should employ longitudinal, multi-method, or experimental designs to test the temporal ordering of variables and strengthen causal inference. Incorporating objective behavioral indicators (e.g., smartphone use logs) and multi-source assessments could also help reduce potential common-method bias and provide a more robust understanding of these relationships.

These findings should also be interpreted with potential contextual influences in mind. Beyond the observed mediational associations, future research could explore possible moderating factors that may shape these relationships. For instance, gender and academic year might influence students’ smartphone usage patterns and self-esteem development. Because male students constituted the majority of the present sample, gender-specific experiences may have contributed to the observed associations. Future studies should therefore aim for more balanced samples to examine whether these relationships vary across genders and academic stages. Moreover, levels of social support may buffer the adverse correlates of smartphone addiction with professional identity and life satisfaction, while psychological capital and personality traits (e.g., self-control, optimism) could moderate or mitigate these associations. Incorporating multi-level and interactional variables in future designs would help clarify under what conditions smartphone use is most strongly related to well-being outcomes and offer a more nuanced understanding of these correlational patterns.

### 5.1 Theoretical implications

This study offers three key theoretical contributions:

First, it expands the theoretical understanding of the mechanisms underlying self-esteem development. While previous research has primarily focused on the influence of personality traits, prosocial behaviors, or family background on self-esteem [39,40], the present findings highlight professional identity and meaning in life as two interrelated psychological correlates of self-esteem. By examining their associational roles in the link between smartphone addiction and self-esteem, this study contributes to a more multidimensional and integrative framework for understanding how contextual and psychological factors may co-vary in shaping students’ self-evaluative systems.

Second, the study deepens theoretical inquiry into the impact pathways of smartphone addiction. Prior work has mainly examined its effects on emotional states and academic performance [41,42]; the present work explores how smartphone dependency co-occurs with variations in self-related constructs such as professional identity and purpose in life. This provides an associational perspective on how digital engagement and self-evaluation may interact, thereby refining the theoretical boundary between media psychology and self-concept research.

Third, this study contributes to the conceptual refinement of professional identity. Whereas previous studies primarily treated professional identity as an outcome, our findings position it as a linking construct between technology-related behavior and self-esteem. This approach underscores the bidirectional and contextual nature of professional identity development, particularly in pre-service teacher populations. Although causal direction cannot be established, the observed relationships suggest that professional identity may play a bridging role in how individuals’ digital habits and self-evaluations are related. This insight invites further longitudinal and experimental research to verify these preliminary patterns and to clarify the temporal dynamics among digital behavior, identity, and self-esteem.

### 5.2 Practical implications

This study found that higher levels of smartphone addiction were associated with lower self-esteem among Physical Education students, with professional identity and meaning in life emerging as indirect correlates in this relationship. Given the cross-sectional design and modest effect sizes, the following recommendations should be interpreted as preliminary, context-sensitive directions rather than prescriptive interventions.

Firstly, digital media literacy education should be incorporated into university-based mental health programmes. Since excessive smartphone use can undermine self-esteem and meaning in life, universities should include a digital media literacy module in their mental health curriculum. Specific measures could include offering specialised courses or lectures, such as ‘Digital Life and Mental Health’, delivered by psychologists or media researchers, to explain the cognitive, emotional and behavioural effects of smartphone use. Other measures could include introducing skill-based training in class, such as case analyses and role-play activities, to equip students with strategies for managing digital addiction and online social pressure, as well as organising experiential reflection activities, such as ‘No-Phone Days’ or ‘Digital Detox Weeks’, in which students could record and reflect on their usage patterns. Such initiatives could strengthen students’ ability to filter information, evaluate content and regulate emotions, helping them to gradually develop healthier smartphone habits and greater resilience when facing digital challenges.

Secondly, develop intervention programmes focused on enhancing self-esteem. Self-esteem is a vital psychological resource that helps students adapt and develop professionally. As this study found that professional identity and meaning in life play central roles in shaping self-esteem, universities should design structured programmes to foster its development. Possible approaches include elective courses on self-esteem and career growth covering topics such as emotion regulation, a growth mindset and self-acceptance, as well as group-based workshops aimed at enhancing self-esteem using methods such as group discussions, positive emotion journaling and mindfulness practice. Peer support groups could also be organised within classes or student associations to provide emotional support and constructive feedback. Additionally, universities should cultivate a positive campus culture through themed events and recognition programmes that encourage students to explore their personal values, experience a sense of meaning and strengthen their self-esteem.

Third, strengthen career value education and professional identity development among students majoring in Physical Education. Because professional identity mediates the link between smartphone addiction and self-esteem, it should be emphasized throughout the training process of preservice PE teachers. Specific measures may include: incorporating scenario-based teaching activities, such as real classroom simulations, to help students experience the responsibilities and roles of PE teachers; requiring students to write reflective journals on professional identity after teaching practice or internships, followed by group discussions; establishing mentorship programs in which experienced teachers guide students in career planning and psychological adjustment; and inviting outstanding in-service PE teachers and educational experts to share experiences and engage in career dialogues, thereby reinforcing students’ sense of identity, mission, and pride in the profession.

In summary, initiatives targeting digital literacy, value construction, and identity development may contribute to students’ well-being and professional growth. However, these implications should be viewed as exploratory and correlational, providing directions for program design rather than direct evidence of intervention efficacy. Universities can use these insights to inform the development of balanced mental health and identity support systems appropriate for the digital era.

### 5.3 Limitations and future research directions

Although this study provides preliminary correlational evidence regarding the psychological mechanisms linking smartphone addiction and self-esteem among Physical Education students through professional identity and meaning in life, several limitations remain that should be acknowledged and addressed in future research.

Firstly, the study employed a cross-sectional survey design, collecting data at a single time point. While significant associations were observed among smartphone addiction, self-esteem, and the proposed mediators, this design precludes conclusions about temporal precedence or causality. Reverse associations (e.g., low self-esteem leading to greater smartphone use) and unmeasured confounding variables may also exist. Future studies should therefore adopt longitudinal or multi-wave designs to examine the stability and sequencing of these relationships over time. Experimental or quasi-experimental approaches could further explore causal processes by manipulating variables such as media-use restrictions or interventions targeting professional identity, thereby enhancing causal inference.

Secondly, as the sample for this study was drawn exclusively from Physical Education students, the generalisability of the findings is limited due to the homogeneity of the participants’ academic backgrounds. Students from different disciplines may experience different academic demands, have different perceptions of their professional roles, and exhibit different behaviours regarding their use of digital media. These factors could influence the structure and strength of the relationship between smartphone addiction and self-esteem. Therefore, future research should recruit more heterogeneous samples spanning multiple disciplines and conduct cross-major comparative analyses to improve the applicability and generalisability of the proposed model. In addition, the current findings should be interpreted with caution and not overgeneralized beyond comparable Physical Education student populations or similar institutional contexts.

Thirdly, the sample in this study was imbalanced in terms of gender, with 74.96% of participants being male. This feature may limit the generalizability of the findings. However, this imbalance reflects the demographic reality of PE teacher education programs in China, where male students are typically overrepresented. Nevertheless, prior studies suggest that gender differences may influence patterns of smartphone use, the development of self-esteem, and broader psychological adjustment. Therefore, future research should aim to achieve a more balanced gender composition where feasible and further examine whether gender moderates the pathways linking smartphone addiction, professional identity, meaning in life, and self-esteem.

In addition, this study only collected data from universities in a single geographic region, without accounting for potential influences of regional cultural differences, variations in educational resources, and contextual social factors. This may constrain the broader applicability of the findings. Future studies should expand the geographic scope by including participants from diverse regions and a variety of institutions (e.g., teacher training universities, comprehensive universities, and sports institutes). This would enable regional comparisons to explore how cultural and contextual factors may moderate the associations among smartphone addiction, self-esteem, and the proposed mediators.

In summary, future research should adopt more rigorous and diverse methodologies, such as multi-wave longitudinal tracking, multi-group comparative designs and mixed methods approaches. These efforts would enhance the theoretical robustness of the proposed framework and offer more nuanced empirical guidance for psychological interventions and educational practices aimed at university students.

## 6 Conclusions

Grounded in SDT and SCT, this study constructed a chain-mediated model to examine the associational relationships among smartphone addiction, professional identity, meaning in life, and self-esteem among students majoring in Physical Education. The main findings are as follows:

(1) Smartphone addiction was negatively associated with self-esteem among Physical Education students.
(2) Professional identity and meaning in life each serve as independent mediators in the relationship between smartphone addiction and self-esteem.
(3) These two mediators also form a significant chain-mediating pathway connecting smartphone addiction to self-esteem.

Together, these findings suggest that higher levels of smartphone addiction may coincide with lower self-esteem, partly through weaker professional identity and diminished meaning in life. Given the cross-sectional, observational design and modest effect sizes, the present results should be interpreted as associational and exploratory rather than causal. The study offers preliminary correlational evidence that contributes to theoretical understanding of how digital media use relates to self-evaluative processes in university students and indicates promising directions for future research and practice aimed at supporting the mental health and professional identity development of pre-service PE teachers.
